# Are disorders of consciousness ‘dis’connection or ‘dys’connection syndromes?

**DOI:** 10.1093/braincomms/fcad328

**Published:** 2023-12-05

**Authors:** Brian L Edlow, Marcello Massimini

**Affiliations:** Center for Neurotechnology and Neurorecovery, Department of Neurology, Massachusetts General Hospital and Harvard Medical School, Boston, MA 02114, USA; Athinoula A. Martinos Center for Biomedical Imaging, Massachusetts General Hospital, Charlestown, MA 02129, USA; Department of Biomedical and Clinical Sciences, University of Milan, Milan 20157, Italy; IRCCS, Fondazione Don Carlo Gnocchi Onlus, Milan 20148, Italy

## Abstract

This scientific commentary refers to ‘Functional hub disruption emphasizes consciousness recovery in severe traumatic brain injury’, by Oujamaa *et al*. (https://doi.org/10.1093/braincomms/fcad319).

**This scientific commentary refers to ‘Functional hub disruption emphasizes consciousness recovery in severe traumatic brain injury’, by Oujamaa *et al*. (https://doi.org/10.1093/braincomms/fcad319).**

Network-based models have become the predominant approach for studying human consciousness and its disorders.^1^ With the advent of neuroimaging and electrophysiological tools that map the brain’s structural connections and functional dynamics,^2^ the network-based approach is rapidly replacing the lesion localization model that predominated for most of the 20^th^ century.^3^ Consistent with conceptual models,^4^ a growing body of empirical evidence points to brain network connectivity properties—specialization and integration—as key elements supporting human consciousness.^5^

It is in this historical context that Oujamaa *et al*.^6^, in their article in this issue of *Brain Communications*, provide new insights into the connectivity properties of network nodes that underly disorders of consciousness (DoC) and enable recovery from DoC in patients with severe brain injuries. In 34 patients with severe traumatic brain injury (TBI) and 20 healthy controls, the authors performed resting-state functional MRI (rs-fMRI) to measure nodal connectivity using graph theoretical analysis. A key innovation of the study design is longitudinal assessment, with rs-fMRI scans performed at discharge from the intensive care unit (ICU) and at discharge from subacute rehabilitation, providing new opportunities to probe network dynamics during the first three months of recovery.

In complementary cross-sectional and longitudinal analyses, the authors measured changes in nodal topology, defined by connectivity to local neighbours (i.e. nodal specialization) and to distant nodes (i.e. nodal integration). These nodal connectivity properties were quantified as the hub disruption index (HDI), an aggregate measure of how an individual patient’s hubs compare to those of healthy, conscious controls. The authors observed that alterations in HDI were present at discharge from the ICU in patients who remained in the minimally conscious state, as compared to those who had fully recovered consciousness. Yet even in patients who fully recovered consciousness by the time of rehabilitation discharge, nodal topology had not completely normalized, as their HDI measurements still differed from those of healthy controls. Collectively, these cross-sectional and longitudinal results indicate that nodal connectivity hubs were converted to ‘non-hubs’ in unconscious patients, and hub connectivity properties remained altered even when consciousness re-emerged.

From a neuroscientific perspective, the disruption of network hubs in patients with DoC at ICU discharge is expected and consistent with prior network hub analyses in this population.^7^ Indeed, there is growing evidence that disruption of hub node connectivity is a common pathway of pathogenesis in multiple neurological disorders.^8^ Yet Oujamaa *et al*.^6^ found that hub disruption coexisted with hub hyperconnectivity, suggesting that DoC may be a ‘dys’connection syndrome, not just a ‘dis’connection syndrome. Whereas disconnection syndromes are characterized by decreased functional integration of brain networks, dysconnection syndromes are characterized by abnormal functional specialization and integration, with coexistence of local hyperconnectivity and global hypoconnectivity.^9^

Additional evidence for conceptualizing DoC as a dysconnection syndrome emerges from the longitudinal finding that HDI measures evolved in parallel with recovery of consciousness. Despite decades of work dedicated to developing network-based biomarkers that predict long-term recovery,^1^ few studies have performed longitudinal analyses of how these biomarkers track re-emergence of consciousness. By demonstrating that hub hypo- and hyperconnectivity patterns both begin to normalize during the first three months of recovery, the authors strengthen the mechanistic link between hub connectivity and human consciousness.

From a clinical standpoint, perhaps the most impactful finding is that patients who recover consciousness have different hub connectivity patterns than do healthy controls. This intriguing observation suggests that the full, normative set of connections in the human connectome is not necessary for consciousness and that functional readjustments play a key role in re-emergence of consciousness after severe brain injury. Accordingly, prognostic models that incorporate connectivity data should account for the possibility that patients may have positive long-term outcomes even when brain network hubs are hypo- or hyperconnected in the ICU. Clinicians should similarly consider the possibility that brain networks with abnormal functional connectivity may be physiologically amenable to therapeutic modulation.^1^

Prior fMRI studies have demonstrated similar patterns of hypo- and hyperconnectivity after TBI, stroke and multiple sclerosis, with relevance to predicting cognitive impairment.^10,11^ Although the neural mechanisms of these alterations remain elusive, recent rodent studies combining electrophysiological and fMRI recordings point to a relationship between the intrusion and propagation of EEG slow waves and functional network abnormalities, including hyperconnectivity.^12^ Similarly, in patients with DoC^13^ and stroke,^14^ cortical sleep-like slow waves are associated with loss of network complexity, providing evidence for local hyperconnectivity (i.e. loss of specialization) and global hypoconnectivity (i.e. loss of integration). Future studies should investigate the spatial relationship between cortical slow waves and nodal hub alterations, as measured by the HDI.

The intriguing findings from Oujamaa and colleagues thus help set the course for the next decade of brain connectivity studies in the field of DoC. From a mechanistic standpoint, are DoC disconnection syndromes characterized by global hypoconnectivity, or as the present results suggest, are DoC more appropriately characterized as dysconnection syndromes characterized by hypo- and hyperconnectivity? From a clinical standpoint, functional connectome mapping with rs-fMRI is already endorsed by clinical guidelines,^15^ but recommendations about which connectivity biomarkers are most relevant to diagnosis, prognosis and therapy selection are lacking. The present study suggests that the HDI biomarker has translational potential in future clinical trials and in the ongoing effort to improve care for patients with DoC.
